# Oncogenic transformation of mesenchymal stem cells decreases Nrf2 expression favoring *in vivo* tumor growth and poorer survival

**DOI:** 10.1186/1476-4598-13-20

**Published:** 2014-02-03

**Authors:** Juan M Funes, Stephen Henderson, Rachel Kaufman, James M Flanagan, Mathew Robson, Barbara Pedley, Salvador Moncada, Chris Boshoff

**Affiliations:** 1 UCL Cancer Institute, University College London, Paul O’Gorman Building, Huntley Street, London WC1E 6BT, UK; 2 Wolfson Institute for Biomedical Research, University College London, Cruciform Building, Gower Street, London WC1E 6BT, UK; 3 Current address: Department of Oncology, Faculty of Medicine, Institute of Reproductive and Developmental Biology, Hammersmith Hospital, Imperial College London, London W12 0NN, UK; 4 Current address: Breast Cancer Clinical Research Unit, Spanish National Cancer Research Centre (CNIO), Madrid 28029, Spain

**Keywords:** Antioxidants, Nrf2, oncogenes, ROS, survival, HIF-1α

## Abstract

**Background:**

The transcription factor Nrf2 is a key regulator of the cellular antioxidant response, and its activation by chemoprotective agents has been proposed as a potential strategy to prevent cancer. However, activating mutations in the Nrf2 pathway have been found to promote tumorigenesis in certain models. Therefore, the role of Nrf2 in cancer remains contentious.

**Methods:**

We employed a well-characterized model of stepwise human mesenchymal stem cell (MSC) transformation and breast cancer cell lines to investigate oxidative stress and the role of Nrf2 during tumorigenesis. The Nrf2 pathway was studied by microarray analyses, qRT-PCR, and western-blotting. To assess the contribution of Nrf2 to transformation, we established tumor xenografts with transformed MSC expressing Nrf2 (n = 6 mice per group). Expression and survival data for Nrf2 in different cancers were obtained from GEO and TCGA databases. All statistical tests were two-sided.

**Results:**

We found an accumulation of reactive oxygen species during MSC transformation that correlated with the transcriptional down-regulation of antioxidants and Nrf2-downstream genes. Nrf2 was repressed in transformed MSC and in breast cancer cells via oncogene-induced activation of the RAS/RAF/ERK pathway. Furthermore, restoration of Nrf2 function in transformed cells decreased reactive oxygen species and impaired *in vivo* tumor growth (*P* = 0.001) by mechanisms that included sensitization to apoptosis, and a decreased hypoxic/angiogenic response through HIF-1α destabilization and VEGFA repression. Microarray analyses showed down-regulation of Nrf2 in a panel of human tumors and, strikingly, low Nrf2 expression correlated with poorer survival in patients with melanoma (*P* = 0.0341), kidney (*P* = 0.0203) and prostate (*P* = 0.00279) cancers.

**Conclusions:**

Our data indicate that oncogene-induced Nrf2 repression is an adaptive response for certain cancers to acquire a pro-oxidant state that favors cell survival and *in vivo* tumor growth.

## Background

An increase in reactive oxygen species (ROS) is a common biochemical property of cancer cells [1,2]. However, excess ROS also induce senescence, cell cycle arrest and apoptosis [3], indicating that redox homeostasis is tightly regulated in tumor cells. To offset excess ROS cells have developed regulatory mechanisms, including the induction of antioxidant enzymes and/or the activation of redox buffering systems such as glutathione. The transcription factor Nrf2 (NFE2L2) plays a crucial role in the cellular defense against oxidative stress through its ability to induce the expression of antioxidant and detoxification genes [4,5]. Under basal conditions, Nrf2 is bound to its inhibitor Keap1 and targeted for degradation by the proteasome pathway [6,7]. Upon certain stress conditions, Nrf2 is released from the inhibitory complex and translocates to the nucleus where it binds antioxidant response elements (ARE) in the promoter regions of its target genes [8,9]. Among these genes are NAD(P)H:quinone oxidoreductase 1 (NQO1), heme oxygenase 1 (HO-1), members of the glutathione S-transferase (GST) family and genes involved in NADPH generation and glutathione biosynthesis [5,10-13].

Activation of the Nrf2-ARE pathway has been proposed as a potential strategy to prevent cancer because of its ability to suppress genotoxic insults by inducing antioxidants and detoxifying enzymes [14-16]. In this regard, *nrf2-/-* mice are more susceptible to chemically-induced cancer [17-20], and Nrf2-deficiency has been suggested to favor metastasis [21]. However, Nrf2 activation has also been proposed to play a role in cancer evolution [22-26], and induction of Nrf2 pathway due to genetic variants in Keap1 or Nrf2 might predispose to cancer [27-30]. Therefore, the role of Nrf2 in cancer is contentious.

Here we employed a previously well-characterized model of human mesenchymal stem cell (MSC) stepwise transformation [31] to mechanistically investigate changes in ROS levels during tumorigenesis. We found an accumulation of ROS during MSC transformation that correlated with the transcriptional down-regulation of antioxidants and ARE-containing genes. Moreover, Nrf2 expression was repressed in transformed MSC and breast cancer cells via activation of RAS/RAF/ERK pathway, and restoration of Nrf2 levels in transformed MSC induced the cellular antioxidant response and impaired *in vivo* tumor growth through mechanisms involving sensitization to apoptosis and destabilization of HIF-1α. Microarray comparison studies showed that expression of Nrf2 is down-regulated in a panel of human tumors, and lower expression of Nrf2 is associated with a poorer outcome in patients with melanoma, kidney and prostate cancers. Overall our results indicate that defects in the cellular antioxidant capacity contribute to ROS accumulation during transformation, and that oncogene-induced Nrf2 repression is an adaptive response for certain cancer cells to acquire a pro-oxidant state that favors cell survival and tumor growth.

## Results

### *In vitro* transformation of human MSC leads to an increase in intracellular ROS that contributes to the transformed phenotype

**Figure 1:**
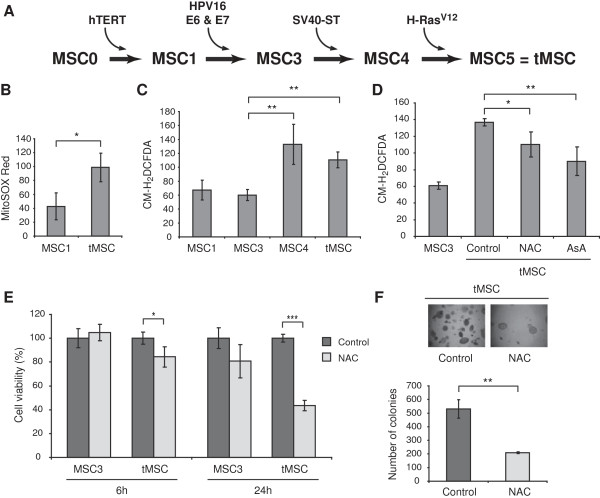
**Transformation of human MSC leads to an increase in intracellular ROS that contributes to the transformed phenotype. (A)** Schematic diagram of MSC step-wise transformation. Cell lines were named according to the number of oncogenic 'hits’ introduced by sequential retroviral transduction. MSC0 are primary MSC, MSC1 are MSC0 expressing hTERT, MSC3 are MSC1 expressing E6 and E7 from HPV-16, MSC4 are MSC3 expressing ST antigen from SV40, and MSC5 are transformed MSC (tMSC) obtained after expression of H-Ras^V12^ in MSC4. **(B)** Intracellular ROS concentration measured by flow cytometry after staining with MitoSOX Red. ROS amounts are presented as the geometrical mean fluorescence intensity (± SD) from at least three experiments. **(C)** Intracellular ROS concentration of MSC expressing different oncogenes measured by flow cytometry after staining with CM-H_2_DCFDA. ROS amounts are presented as the geometrical mean fluorescence intensity (± SD) from at least three experiments. **(D)** ROS production in tMSC measured by CM-H_2_DCFDA staining after 4 hours treatment with 2.5 mM N-acetyl-L-cysteine (NAC) or 50 μM ascorbic acid (AsA). ROS levels produced by MSC3 are also shown. **(E)** Cell viability after 6 and 24 hours treatment with 2.5 mM NAC or vehicle control was addressed by using CellTiter AQueousOne Solution Cell Proliferation Assay (Promega) following the manufacturer’s instructions. A representation of three different experiments is shown and data were calculated from the average of four replicate wells. Results are shown as percentage of viable cells referred to control cells (MSC3) treated with vehicle control. **(F)** Soft agarose transformation assay of untreated and 2.5 mM NAC treated tMSC. Colonies were photographed at x40 magnification (top) and counted in triplicate wells (bottom) after 12 days in culture. *P* values are *P < 0.05, **P < 0.005 and ***P < 0.0005.

To investigate changes in ROS levels during tumorigenesis, we employed a previously developed stepwise transformation model of human MSC (Figure 1A) [31]. Briefly, primary MSC (MSC0) were sequentially infected with the human telomerase (hTERT) gene (MSC1) and the oncoproteins E6 and E7 from HPV-16 (MSC3). The expression of these genes led to cellular immortalization and to the inactivation of p53 and pRB tumor suppressors. The additional expression of ST antigen from SV40 (MSC4) and oncogenic H-Ras^V12^ (MSC5) has been shown to induce transformation in other human cells [32]. MSC expressing these five genes acquired full transformed features as showed by their ability to induce tumors in nude mice [31]. Therefore, MSC5 or transformed MSC were named thereafter tMSC. To determine the production of ROS during MSC transformation, we measured ROS levels by flow cytometry after cell staining with MitoSOX Red, a dye commonly used for the detection of mitochondrial free radical superoxide $\left(O_{2}^{\;•\;-}\right)$. This staining led to more than two fold increase in the fluorescence intensity of tMSC when compared with immortal MSC1 (Figure 1B). To delineate the step during *in vitro* transformation where increased ROS occur, we compared the fluorescence intensity of MSC expressing different oncogene combinations after staining with CM-H_2_DCFDA, a dye that detects different types of ROS including hydrogen peroxide (H_2_O_2_). While immortal MSC1 produced similar amounts of ROS to MSC3, the additional expression of ST (MSC4) and H-Ras^V12^ (tMSC) led to a significant increase in ROS production (Figure 1C). Since increased ROS have been shown to promote tumor development and progression, we next investigated whether ROS scavenging by antioxidants affected the viability and the transforming capabilities of tMSC. Treatment with N-acetyl-L-cysteine (NAC) or ascorbic acid diminished the accumulation of ROS in tMSC (Figure 1D). We also found that NAC compromised the viability of tMSC, but not that of immortal MSC3 (Figure 1E) or MSC1 (data not shown). Furthermore, NAC treatment impaired *in vitro* transformation of tMSC measured by colony formation in soft agarose (Figure 1F), suggesting that a certain threshold of intracellular ROS levels is required to maintain the transformed phenotype of MSC.

### Transformation of MSC induces transcriptional down-regulation of antioxidant genes

**Figure 2:**
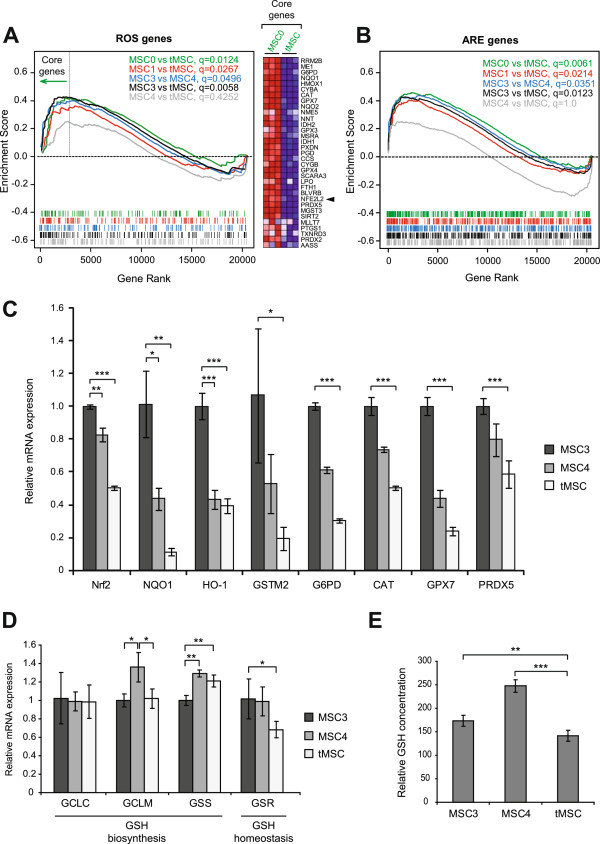
**Transformation of MSC induces transcriptional down-regulation of antioxidant genes. (A)** The enrichment of genes involved in ROS metabolism was addressed by GSEA using gene expression microarray data from different paired cell lines (q values from different analysis are shown). The Y-axis plots the enrichment score and the X-axis is the rank of genes within the ROS list from up-regulated to down-regulated. The barcode below shows the rank position of individual ROS genes within the list. Arrow indicates ROS genes identified as the core-enriched genes by GSEA when MSC0 was compared with tMSC. Heatmap at the right panel shows relative expression of genes found within this core-enriched gene region from cell lines MSC0 and tMSC, where multiple probes per gene were averaged. Results were obtained by performing array experiments in triplicate (columns). Red indicates up-regulation, and blue down-regulation from the mean. Arrowhead indicates Nrf2 (NFE2L2). **(B)** GSEA shows the enrichment of ARE-containing genes for different MSC paired lines. **(C)** Relative mRNA expression of selected genes involved in antioxidant response measured by qRT-PCR. These genes include Nrf2, NAD(P)H dehydrogenase quinone 1 (NQO1), heme oxygenase 1 (HO-1), glutathione S-transferase M2 (GSTM2), glucose-6-phosphate dehydrogenase (G6PD), catalase (CAT), glutathione peroxidase 7 (GPX7) and peroxiredoxin 5 (PRDX5). **(D)** Relative mRNA expression of genes involved in glutathione biosynthesis (GCLC, GCLM, and GSS) and homeostasis (GSR). **(E)** Levels of intracellular reduced glutathione (GSH) presented as relative luciferase units per microgram of protein x10^5^. The result shows the average of 2 experiments performed with at least 4 replicates each. *P* values are *P < 0.05, **P < 0.005 and ***P < 0.0005.

To investigate potential mechanisms for increased ROS in tMSC we exploited gene expression microarray data previously generated in our laboratory [31]. Gene Set Enrichment Analysis (GSEA) [33] performed with a compilation of genes that included a previously published list of genes involved in ROS metabolism [34] (Additional file 1: Table S1) showed an enrichment of ROS-related genes (q < 0.05) in those cell lines expressing fewer number of oncogenes, except for the comparison between MSC4 and tMSC, where no significant enrichment was observed (q = 0.4252) (Figure 2A, left panel). Many genes involved in the antioxidant response, including Nrf2 (NFE2L2), were found within the group of genes showing most deregulated expression when MSC0 was compared with tMSC (Figure 2A, right panel). Since Nrf2 binds ARE-containing sequences we used a previously generated list of genes known to contain ARE in their promoters (a full list in Supplementary Table Three from [35]) and performed GSEA with different pairs of MSC lines. This analysis showed an enrichment of ARE-containing genes (q < 0.05) in those cell lines expressing fewer number of oncogenes, except for the comparison between MSC4 and tMSC that showed no enrichment (q = 1) (Figure 2B).

We focused on the last steps during MSC transformation where significant changes in intracellular ROS levels were found (from MSC3 to MSC4 and tMSC). qRT-PCR experiments confirmed down-regulation of Nrf2 and selected antioxidants and ARE-containing genes when tMSC were compared with MSC3 and MSC4 (Figure 2C). One of the most powerful antioxidants and a major redox buffering mechanism in the cell is the glutathione system (GSH/GSSG). Expression of genes involved in glutathione biosynthesis such as glutamate-cysteine ligase catalytic and modifier subunits (GCLC and GCLM), and glutathione synthetase (GSS) fluctuated during the process of MSC transformation (Figure 2D). We also found diminished expression of glutathione reductase (GSR, another Nrf2-down-stream gene) in tMSC, suggesting that inefficient conversion of oxidized glutathione (GSSG) to its reduced form (GSH) occurs in tumor cells (Figure 2D). Concurring with these results, tMSC showed the lowest levels of the active (reduced) form of glutathione (GSH), the form of glutathione able to provide antioxidant power (Figure 2E). Overall, these data indicate that transformation of MSC leads to a global transcriptional down-regulation of the cellular antioxidant program.

### Nrf2 is repressed during cellular transformation via activation of RAS/RAF/ERK pathway

**Figure 3:**
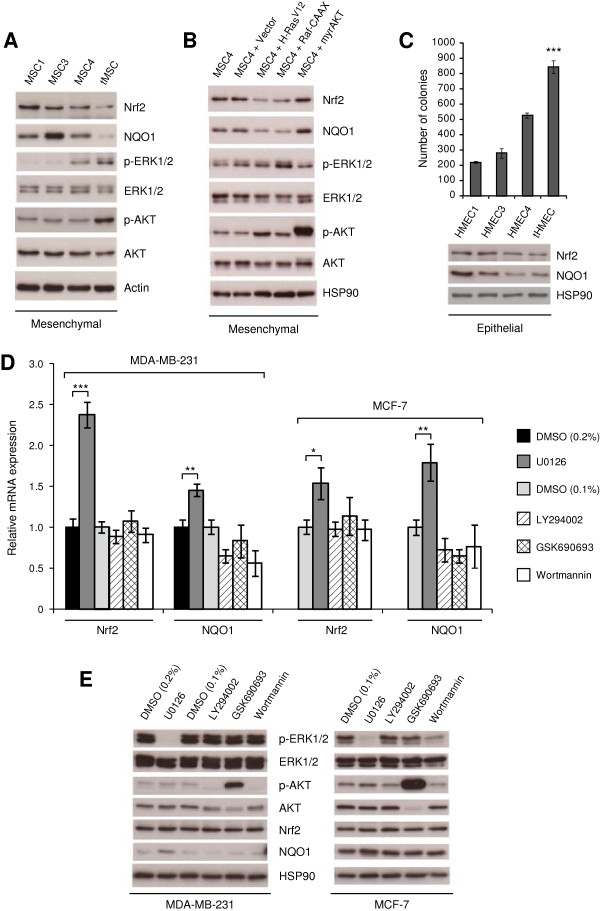
**Nrf2 is repressed during cellular transformation via activation of RAS/RAF/ERK pathway. (A)** Western-blot shows down-regulation of Nrf2 and downstream target NQO1 during transformation of human MSC. Activation of AKT and ERK pathways, confirmed by increased phosphorylation of AKT and ERK1/2 proteins, correlates with decreased expression of Nrf2 and NQO1 in tMSC. **(B)** Expression of constitutive active mutants H-Ras^V12^ and Raf-CAAX, but not myrAKT, leads to Nrf2 and NQO1 down-regulation in non-transformed MSC4. **(C)** Immortal human mammary epithelial cells (HMEC1) were serially transduced with retroviral vectors encoding the genes E6 and E7 from HPV-16 (HMEC3), ST antigen from SV40 (HMEC4), and H-Ras^V12^ (tHMEC). Top panel shows the number of colonies in soft-agarose counted in triplicate wells after two weeks in culture. ***P < 0.0005 with respect to all. Western-blot at the bottom panel shows Nrf2 and NQO1 repression during HMEC transformation. **(D)** qRT-PCR shows that 16 hours treatment with the ERK inhibitor U0126 induces expression of Nrf2 and its downstream gene NQO1 in MDA-MB-231 and MCF-7 cells. However, treatment with the AKT inhibitor GSK690693 or with the PI3K inhibitors LY294002 and wortmannin does not induce Nrf2 expression. DMSO served as a control. **(E)** Western-blots show inactivation of ERK and PI3K/AKT pathways following 16 hours treatment with inhibitors. Membranes were probed with rabbit polyclonal antibodies for Nrf2, total ERK1/2, phosphorylated ERK1/2 (p-ERK1/2 (Thr202/Tyr204)), total AKT, phosphorylated AKT (p-AKT (Ser473)), and monoclonal antibodies for NQO1. Actin and Hsp90 were used as a loading control. *P* values are *P < 0.05, **P < 0.005 and ***P < 0.0005.

Western-blot experiments confirmed suppression of Nrf2 expression and its downstream target NQO1 that correlated with ST- and H-Ras^V12^-induced activation of ERK and AKT pathways (Figure 3A). To investigate the mechanism of Nrf2 repression during transformation, we focused in the last transformation step where the more pronounced down-regulation of Nrf2 and ARE-containing genes occurred. We studied the roles of RAS and some RAS-downstream effectors by expressing constitutive active mutants of H-RAS (H-Ras^V12^), RAF-1 (Raf-CAAX), and AKT (myrAKT) in immortal MSC4. We found that activation of RAS and RAF, but not AKT, led to decreased expression of Nrf2 and NQO1 (Figure 3B).

Recent reports showed that Nrf2 expression was decreased in certain human breast cancer cells and breast tumors when compared with normal mammary epithelial cells or normal breast tissue [36,37]. Interestingly, we found a reduction in Nrf2 and NQO1 expression when normal human mammary epithelial cells (HMEC) were transformed using the same oncogenic elements that we employed to transform MSC (Figure 3C), suggesting that this mechanism for Nrf2 regulation is not restricted to adult MSC. Next we used chemical inhibitors to address whether Nrf2 expression is transcriptionally regulated via ERK or PI3K/AKT pathways in the breast cancer cell lines MDA-MB-231 and MCF-7. While cell survival was not affected by the concentration of inhibitors used in this assay (Additional file 2: Figure S1), treatment with the ERK inhibitor U0126 led to a significant increase in the transcription of Nrf2 and NQO1 (Figure 3D). However, inhibition of AKT with GSK690693, or PI3K with LY294002 and wortmannin did not induce expression of Nrf2 nor NQO1 (Figure 3D). The effect of these inhibitors on ERK and PI3K/AKT pathways is shown in Figure 3E, where a modest but consistent activation of the Nrf2 pathway could be detected following only 16 hours treatment with U0126. Overall our data indicate that the RAS/RAF/ERK pathway mediates Nrf2 repression in these cancer cells.

Nrf2 activity was found suppressed in tumor cells due to increased expression of the ubiquitin ligase Cul3 that, together with Keap1, targets Nrf2 for degradation by the proteasome [36]. However, expression of Keap1 (which is wild type in MSC, data not shown) and Cul3 did not increase in transformed MSC (Additional file 3: Figure S2).

### Nrf2 protein stabilization by means of tert-butylhydroquinone (TBHQ) impairs MSC transformation

**Figure 4:**
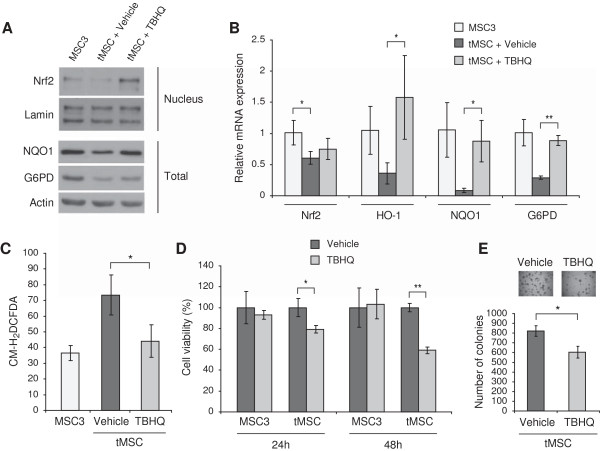
**Chemical stabilization of Nrf2 induces antioxidant enzymes, reduces ROS, and impairs MSC transformation. (A)** Nuclear and total cellular extracts of MSC3 and tMSC treated with 10 μM TBHQ or vehicle for 48 hours were analyzed for the expression of Nrf2, NQO1 or G6PD by immunoblotting. Rabbit monoclonal antibodies were used for Nrf2, and lamin and actin were used as loading controls. **(B)** qRT-PCR of Nrf2 and Nrf2-dowstream genes such as HO-1 and NQO1 was performed in tMSC treated for 48 hours with 10 μM TBHQ or vehicle control. Non-transformed MSC3 were used as a control. **(C)** Intracellular ROS concentration measured by CM-H_2_DCFDA staining in tMSC treated with or without 10 μM TBHQ for 48 hours. MSC3 ROS levels are also shown. ROS amounts are presented as the geometrical mean fluorescence intensity (± SD) from at least three experiments. **(D)** Cell viability after treatment with 10 μM TBHQ or vehicle control. A representation of three independent experiments is shown and data were calculated from the average of four replicate wells. Results are shown as percentage of viable cells referred to control cells (MSC3) treated with vehicle control. **(E)** Soft agarose colonies of tMSC treated with vehicle only or with 10 μM TBHQ were photographed at x40 magnification after 12 days in culture (top) and counted in triplicate wells (bottom). *P* values are *P < 0.05 and **P < 0.005.

To investigate whether Nrf2 down-regulation contributes to increased ROS, we induced Nrf2 in tMSC by TBHQ, a chemical that stabilizes Nrf2 protein by impairing its proteasomal degradation [38,39]. Treatment with TBHQ stabilized Nrf2 (Figure 4A), induced antioxidants (Figure 4A and B) and reduced ROS levels in tMSC (Figure 4C). We next tested whether ROS scavenging by TBHQ affected the transforming capabilities of tMSC. TBHQ significantly impaired the growth of tMSC, but not that of immortal MSC3 (Figure 4D). Furthermore, treatment with TBHQ decreased anchorage-independent growth of both tMSC and tHMEC measured by soft agarose colony formation (Figure 4E and Additional file 4: Figure S3, respectively). These results suggest that loss of Nrf2 expression contributes to both accumulation of intracellular ROS, and to MSC *in vitro* transformation.

### Restoration of Nrf2 expression in tMSC induces the cellular antioxidant response and impairs *in vivo* tumor growth

**Figure 5:**
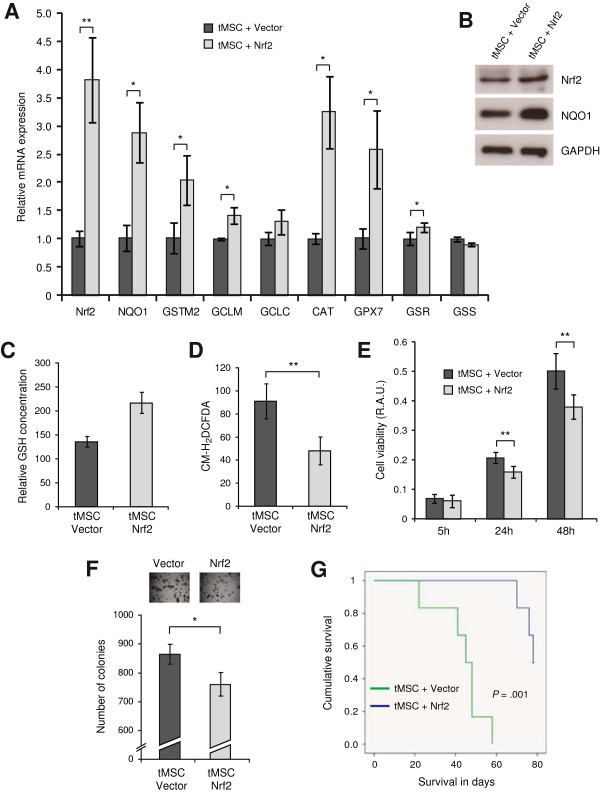
**Restoration of Nrf2 expression in transformed MSC induces an antioxidant response and impairs tumor growth. (A)** Relative mRNA expression of Nrf2 and selected antioxidants measured by qRT-PCR in tMSC over-expressing either empty vector or Nrf2. These genes include NQO1, GSTM2, glutamate-cysteine ligase modifier and catalytic subunit (GCLM and GCLC respectively), CAT, GPX7, glutathione reductase (GSR) and glutathione synthetase (GSS). **(B)** Total cell extracts of tMSC expressing either empty vector or Nrf2 were analyzed for expression of Nrf2 and its downstream target NQO1 by immunoblotting. Polyclonal antibodies were used for Nrf2 and GAPDH served as a loading control. **(C)** Levels of intracellular reduced glutathione (GSH) presented as relative absorbance units per microgram of protein x10^5^. The result shows the average of 2 experiments performed with at least 4 replicates each. **(D)** Intracellular ROS levels of tMSC expressing either empty vector or Nrf2, measured by CM-H_2_DCFDA staining. ROS amounts are presented as the geometrical mean fluorescence intensity (± SD) from at least three experiments. **(E)** Growth rate of tMSC expressing empty vector or Nrf2 at 5, 24 and 48 hours. The number of viable cells was expressed as relative absorbance units (R.A.U.). The average of three experiments with at least four replicate wells per cell line and normalized by the amount of protein is shown. **(F)** Soft agarose colonies of tMSC over-expressing empty vector or Nrf2 were photographed after 12 days in culture at x40 magnification (top) and counted in triplicate wells (bottom). **(G)** Comparison of animal survival between the Nrf2 over-expressing group and empty vector group containing six mice each (*P* = 0.001). *P* values are *P < 0.05 and **P < 0.005.

To validate the observed effect of TBHQ in our model, we genetically over-expressed Nrf2 in transformed MSC. tMSC over-expressing Nrf2 exhibited increased transcription of ARE-containing genes and antioxidant enzymes (Figure 5A). Activation of the Nrf2 pathway was confirmed by increased expression of Nrf2 and NQO1 proteins (Figure 5B). Furthermore, tMSC over-expressing Nrf2 showed an increase in the pool of reduced glutathione (Figure 5C) and a decrease in intracellular ROS (Figure 5D). Next, we investigated how Nrf2-mediated reduction in ROS levels affected the transformation capability of tMSC. Over-expression of Nrf2 led to a slight, but significant reduction in tMSC viability (Figure 5E) and soft agarose growth (Figure 5F) when compared with tMSC expressing empty vector. Next we questioned whether these cells could respond differentially when they encounter physiological conditions *in vivo*. Hence we inoculated tMSC over-expressing Nrf2 or empty vector into nude mice. While all mice from the empty vector group showed rapidly growing tumors, only three out of six mice from the Nrf2 group produced tumors, and these after a significantly longer latency (*P* = 0.001) (Figure 5G).

### Nrf2 over-expression sensitizes tMSC to apoptosis and diminishes the angiogenic response by destabilization of HIF-1α and VEGF repression

**Figure 6:**
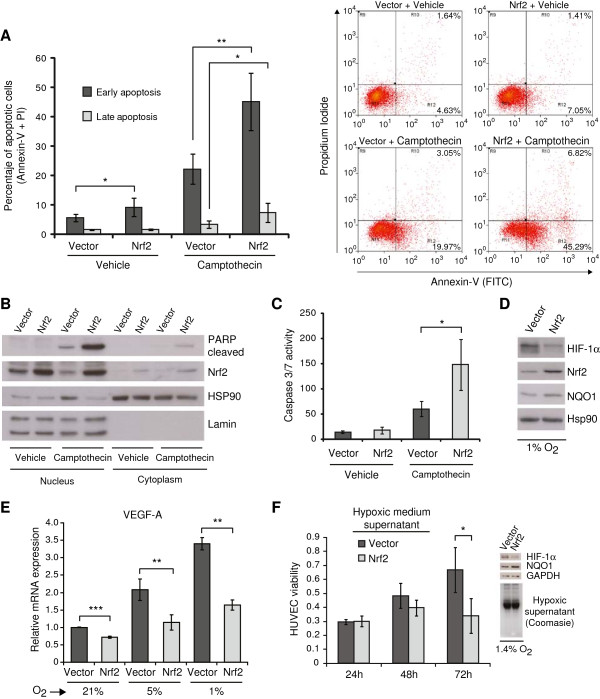
**Nrf2 sensitizes tMSC to apoptosis and diminishes the *in vitro *angiogenic response. (A)** Apoptosis measured by flow cytometry after double staining with Annexin-V (FITC) and Propidium Iodide (PI). Left panel shows the percentage of cells in early apoptosis (positive for FITC) and late apoptosis (positive for FITC and PI) after 24 hours treatment with 5 μM camptothecin. Data show the mean ± S.D. from five independent experiments. Right panel shows the dot plots of a representative experiment. **(B)** Western-blot of nuclear and cytoplasmic extracts showing induction of cleaved PARP protein. Membrane was also probed with mouse monoclonal antibodies against Nrf2. Hsp90 and Lamin served as loading controls. **(C)** Caspase 3/7 activity of cells treated likewise. Results are expressed as relative absorbance units normalized by protein amount. Data show the mean ± S.D. from three independent experiments. **(D)** Western-blot shows destabilization of HIF-1α protein in Nrf2-over-expressing cells grown for 48 hours in hypoxia. Membranes were probed with mouse monoclonal antibodies for HIF-1α, Nrf2 and NQO1. HSP90 served as control. **(E)** qRT-PCR shows the VEGF-A fold induction in normoxic (21% O_2_) and 48 hours hypoxic (5% and 1% O_2_) cells. **(F)** Effect of hypoxic medium supernatant from tMSC over-expressing vector or Nrf2 in HUVEC viability. Supernatants from tMSC grown in hypoxia for 48 hours were mixed with endothelial cell medium at 50% ratio prior to incubation with HUVEC. A representation of three independent experiments shows the viable cells at 24, 48 and 72 hours as relative absorbance units. Western-blot shows the failure of Nrf2-expressing cells to accumulate HIF-1α protein in hypoxia (top-right). Increased NQO1 expression indicates activation of Nrf2 pathway. Equal loading of hypoxic tMSC medium supernatant in HUVEC is shown by coomassie blue staining (bottom-right). *P* values are *P < 0.05, **P < 0.005 and ***P < 0.0005.

Due to the different responses observed *in vitro* and *in vivo*, we challenged the cells to a variety of stressors in order to mimic aspects of the *in vivo* tumor microenvironment. We found that tMSC over-expressing Nrf2 exhibited more apoptotic cells when compared with control cells after double staining with Annexin-V (FITC) and Propidium Iodide (Figure 6A). Furthermore, Nrf2 sensitized cells to apoptosis induced by the DNA-damaging agent camptothecin (an inhibitor of topoisomerase I) as measured by staining with Annexin-V (FITC) and Propidium Iodide (Figure 6A and Additional file 5: Figure S4), by accumulation of cleaved PARP protein (Figure 6B), and by increased caspase 3 and 7 activity (Figure 6C). Likewise, cells over-expressing Nrf2 showed increased cytotoxicity following treatment with the apoptotic inducers etoposide (a topoisomerase II inhibitor) and the ATP-competitive kinase inhibitor staurosporine (Additional file 6: Figure S5).

ROS are implicated in the response to hypoxia through a mechanism involving stabilization of hypoxia-inducible factor 1 (HIF-1α) [40]. Interestingly, tMSC over-expressing Nrf2 were not able to stabilize HIF-1α at 1% O_2_ concentration (Figure 6D). Furthermore, the expression of vascular endothelial growth factor A (VEGFA), an angiogenic HIF-1α-downstream gene, was significantly reduced in Nrf2-expressing cells grown at 21% O_2_ (Figure 6E). VEGFA production was further decreased when Nrf2-expressing cells were grown at 5% and 1% O_2_ concentrations (Figure 6E). Besides, we also found that cells over-expressing Nrf2 in hypoxic conditions showed a significant decreased expression of adrenomedullin (ADM), another HIF-1α-dependent angiogenic and anti-apoptotic gene [41] (Additional file 7: Figure S6).

Angiogenesis depends on the capacity of endothelial cells to proliferate and migrate. We next tested whether viability of human umbilical vein endothelial cells (HUVEC) is affected by conditioned medium from transformed cells over-expressing Nrf2. HUVEC cultured with hypoxic conditioned medium from tMSC expressing Nrf2 showed a significant impairment in viability when compared with HUVEC treated with hypoxic conditioned medium from tMSC expressing empty vector (Figure 6F). This result suggests that loss of Nrf2 expression in tumor cells could facilitate the proliferation of endothelial cells within the tumor microenvironment in conditions when oxygen concentration becomes limited.

### Lower Nrf2 expression is associated with poorer survival in certain cancers

**Figure 7:**
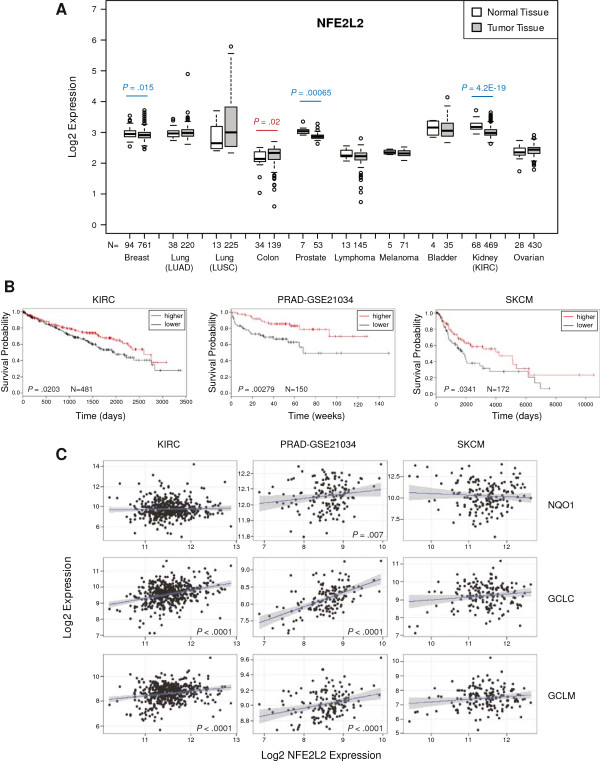
**Decreased Nrf2 expression in certain tumors is associated with poor survival. (A)** The analysis of 2548 tumors versus 304 normal tissues revealed that Nrf2 (NFE2L2) expression is significantly down-regulated in breast, prostate, and kidney (KIRC) tumors (*P* values in blue), and significantly up-regulated in colon cancer (*P* value in red) when compared with normal tissue. However, there are not significant changes in the expression of NFE2L2 when normal tissue is compared with tumors such as bladder, ovarian, lung adenocarcinoma (LUAD), lung squamous cell carcinoma (LUSC), lymphoma and melanoma. Data are presented as log-transformed, and normalized by subtracting the mean across all arrays. The type of tumor and the number of samples analyzed (N) are indicated on the X-axis. These data are taken from TCGA database ( https://genome-cancer.ucsc.edu). **(B)** Low NFE2L2 expression correlated with poorer survival in patients with melanoma (SKCM, *P* = 0.0341, N = 172), kidney (KIRC, *P* = 0.0203, N = 481) and prostate (PRAD-GSE21034, *P* = 0.00279, N = 150) cancers. The Kaplan-Meir survival curves for KIRC, survival in days; PRAD-GSE21034, biochemical recurrence in weeks; and SKCM, survival in days are shown. Samples were categorized as below (lower, black line) or above (higher, red line) median NFE2L2 expression, and the *P* values were calculated from the log rank (Mantel Cox) test. The hazard ratio for lower NFE2L2 expression were KIRC: 1.46 (1.06-2.02, 95% conf. lim.), SKCM: 1.62 (1.03-2.57, 95% conf. lim.), PRAD-GSE21034: 2.74 (1.375-5.34, 95% conf. lim.). **(C)** In the same expression datasets we find that NFE2L2 expression is significantly positively correlated with GCLC and GCLM in KIRC, with GCLC, GCLM and NQO1 in PRAD-GSE21034, but with none of these genes in SKCM (as determined using Spearmans rank correlation). The *P* values are shown for those genes showing a significant correlation.

We next explored whether Nrf2 is differentially expressed between normal and cancer tissues. Microarray comparison studies based on data from the Oncomine database [42] revealed that the majority of tumors showed low levels of Nrf2 expression when compared to normal tissue (Additional file 8: Figure S7). A more comprehensive microarray analysis based on The Cancer Genome Atlas (TCGA) database that included 8 of the 10 most common human malignancies showed that Nrf2 expression was significantly down-regulated in breast (*P* = 0.015), prostate (*P* = 0.00065) and kidney (*P* = 4.2E-19) tumors, with only colon cancer showing up-regulated Nrf2 expression (*P* = 0.02) when compared to normal tissue (Figure 7A). We also found a significant down-regulation in the expression of the Nrf2 downstream genes GCLM, GCLC and NQO1 in breast, prostate and kidney cancer respectively (Additional file 9: Figure S8), suggesting that Nrf2 protein activity might also be reduced in these tumors. Of note, analysis of Keap1 expression in these datasets showed no significant differences between normal and tumors samples, except for lymphoma tumors where Keap1 expression was found up-regulated (*P* = 4.7E-6) when compared to normal tissue (Additional file 10: Figure S9).

Next we investigated whether Nrf2 levels are associated with survival in patients with cancer. Analysis of available survival datasets obtained from GEO and TCGA databases showed that lower expression of Nrf2 (NFE2L2) is associated with a significantly poorer outcome in skin cutaneous melanoma (SKCM) (*P* = 0.0341) and in kidney clear cell carcinoma (KIRC) (*P* = 0.0203) (Figure 7B). Similarly, low Nrf2 expression was associated with biochemical recurrence in prostate cancer (PRAD-GSE21034) (*P* = 0.00279) [43] (Figure 7B), but we found no relation positive or negative to prognosis in any of the other cancers studied (detailed in Additional file 11: Table S2). The analysis of those cancers where we found an association between Nrf2 expression and survival revealed that the mRNA level of Nrf2 was positively correlated to its downstream targets in KIRC and PRAD-GSE21034, but not in SKCM (Figure 7C). These data suggest that the Nrf2 pathway activity can be diminished in those tumors exhibiting low Nrf2 expression.

## Discussion

Intracellular redox homeostasis is altered in cancer, where increased levels of ROS favor a pro-oxidant microenvironment [2]. Here we show that MSC accumulate ROS during oncogenic transformation, and that transformed MSC become oxidative stress-dependent, since treatment with antioxidants decreases ROS levels and impairs their tumorigenic potential. Moreover, the increase in ROS coincides with the down-regulation of genes involved in the cellular antioxidant machinery, including most antioxidant enzymes, genes implicated in glutathione homeostasis, and those involved in the biosynthesis of NADPH. It is believed that a significant amount of the intracellular ROS is produced by mitochondria. However, ROS can also be produced by non-mitochondrial sources such as membrane-bound NADPH oxidases [44]. While the vast majority of the pro-oxidant enzymes in our list of ROS genes (including most of the NADPH oxidases) do not change during MSC transformation (data not shown), microarray and qRT-PCR analysis showed increased expression of NADPH oxidase 4 (NOX4) and aldehyde oxidase 1 (AOX1) during MSC transformation (data not shown). Although the precise contribution of these enzymes to ROS accumulation is unknown and needs further investigation, our data overall suggest that a defective cellular antioxidant system may largely contribute to the high levels of ROS observed during MSC transformation.

We also found that expression of Nrf2 decreased during the process of MSC transformation. Although we cannot rule out the possibility that both ST and oncogenic Ras may interfere with Nrf2 protein stability, we focused our attention on the role of H-Ras^V12^ as it induced the most marked down-regulation of Nrf2 expression. Our data indicate that activation of the RAS/RAF/ERK pathway represses Nrf2 expression and contributes to the diminution of the cellular antioxidant response during MSC transformation. Nrf2 and its downstream target NQO1 were also suppressed in transformed human mammary epithelial cells, indicating that this mechanism for ROS accumulation is not restricted to adult stem cells. These results are in concordance with previous reports where ERK inhibition in the presence of insulin increases ARE-luciferase activity in HL-1 mouse cardiac cells [45], and where the RAS/RAF/ERK pathway was proposed to inhibit Nrf2 in human neuroblastoma cells with Myc amplification [46]. Moreover, analysis of previous microarray studies where investigators have transformed cells *in vitro*[47,48] showed that oncogenic transformation leads to Nrf2 down-regulation in both mouse and human cells (Additional file 12: Figure S10). However, our results are in contrast to those from a report by DeNicola et al. [25] where conditional activation of K-Ras^G12D^ in a mouse model of pancreatic cancer induced the expression of Nrf2 via the RAF pathway. This divergence could be due to the different approach employed to express oncogenes, as H-Ras^V12^ was constitutively expressed in human MSC and breast epithelial cells, whereas K-Ras^G12D^ was conditionally activated in the mouse model. These approaches might elicit quantitative different levels of Ras activity in the target cells, resulting in a different regulatory mechanism for Nrf2 expression. However, rather than super-physiologic expression of Nrf2, we restored Nrf2 levels to that observed in non-transformed MSC, suggesting that our model is relevant to transformation of primary human cells. Other divergences between our work and that from DeNicola et al. are the different species and tumor models studied, as well as the different stage during tumor development. In this regard, oncogenic Ras might induce different biological responses depending on the status of tumor suppressors such as p53 and/or oncogenes such as Myc.

Here we show that Nrf2-mediated induction of the cellular antioxidant response is an efficient strategy to tackle *in vivo* tumor growth in transformed adult stem cells. Mechanistically, we show that Nrf2 sensitizes transformed cells to apoptosis, contrasting with previous reports where Nrf2 was shown to protect from apoptosis and to enhance drug resistance [49,50]. However, our results are in concordance with previous findings where the presence of antioxidants was found to improve the cytotoxic effect of apoptosis-inducing agents [51]. Future studies should address the effects of Nrf2 on the regulation of pro- and anti-apoptotic proteins in transformed MSC.

We also provide evidence linking Nrf2 activation with a reduced angiogenic response under hypoxic conditions. In agreement with findings that ROS may regulate angiogenesis and tumor growth through HIF-1α and VEGF [52], over-expression of Nrf2 in tMSC led to a diminished hypoxic response through destabilization of HIF-1α and reduced VEGFA and ADM expression. These data differ from a report where Nrf2 knockdown by siRNA in human colon cancer cells inhibited tumor growth and led to a reduction in VEGF expression [53]. However, our data suggest that hypoxic conditions could result in a more hostile microenvironment for cells with higher levels of Nrf2.

All these discrepancies add more complexity to the contentious function of Nrf2 during tumorigenesis. Indeed, it has been suggested that the role of Nrf2 in cancer is context-dependent [54]. In this regard, a recent report based on an urethane-induced multistep mouse model of lung cancer has proposed that Nrf2 has the dual role of preventing tumor initiation, but also promoting tumor progression [55]. However our data reveal a tumor suppressor role for Nrf2 since its down-regulation contributes to cellular transformation and *in vivo* tumor growth. Microarray comparison studies support our experimental data, indicating that expression of Nrf2 is down-regulated in many tumors. Moreover, analysis of available survival datasets obtained from GEO and TCGA databases shows that increased Nrf2 expression correlates with better survival in patients with melanoma, kidney and prostate cancers, further supporting our *in vivo* findings where restoration of Nrf2 expression in transformed MSC improved survival.

## Conclusions

Overall our results indicate that defects in the cellular antioxidant capacity contribute to ROS accumulation during transformation, and that oncogene-induced Nrf2 repression is an adaptive response for certain cancer cells that favors *in vivo* tumor expansion and poorer survival. We also show that rescue of Nrf2 function in fully transformed cells is an effective strategy to tackle *in vivo* tumor growth, as Nrf2 expression sensitizes transformed cells to apoptosis and impairs the angiogenic response through destabilization of HIF-1α.

## Methods

### Cell culture and generation of stable cell lines

Culture conditions, retrovirus production and generation of cell lines were previously described [31]. Briefly, primary human MSC previously isolated from the bone marrow of a healthy donor (MSC0) according to institutional guidelines were serially transduced with retroviruses encoding hTERT (MSC1), E6 and E7 from HPV-16 (MSC3), ST antigen from SV40 (MSC4), and H-Ras^V12^ (tMSC). For the generation of tMSC over-expressing Nrf2, we amplified the Nrf2 gene from human cDNA using the following primers: forward (5′-GCGGATCCATGATGGACTTG-3′) and reverse (5′-ACGCGTCGACCTAGTTTTTCTTAACATC-3′). Nrf2 was later cloned into pWZL-hygro and used to infect tMSC where H-Ras^V12^ had been previously introduced with pWZL-blast.

### Detection of intracellular ROS

ROS levels were quantified by staining the cells with MitoSOX Red and CM-H_2_DCFDA dyes (both from Invitrogen, Paisley, UK). After 30 minutes incubation with the dyes at 5 μM final concentration, cells were collected and analyzed by flow cytometry using either a FACSCalibur instrument (BD Biosciences, San Jose, CA) or a CyAN flow cytometer (DAKO, Cambridgeshire, UK). Data were analyzed using either CellQuest V or Summit software. Cell incubation with MitoSOX was performed in serum-depleted media.

### Transformation assays

Soft agarose colony formation by anchorage-independent growth and tumor xenografts were previously described [31]. The animal experiments were conducted in accordance with institutional guidelines under the approved protocols. For the *in vivo* tumor growth experiments, Kaplan Meier survival plots were generated, and from the survival data a log rank (Mantel-Cox) test was used to demonstrate significant differences between groups.

### Antibodies and reagents

The following antibodies were used for immunoblotting: rabbit polyclonal C-20 (Santa Cruz, CA), mouse monoclonal (M01) clone 1 F3 (Abnova, Taiwan), and rabbit monoclonal EP1808Y (Abcam, Cambridge, UK) for Nrf2; Actin was from Calbiochem/MerckMillipore (Watford, UK); NQO1 was from Novus Biologicals (Cambridge, UK); G6PD was from Bethyl (Montgomery, TX); HIF-1α was from BD Biosciences (San Jose, CA); Cleaved PARP, total AKT, phosphorylated AKT (Ser473) (p-AKT), total ERK1/2, phosphorylated ERK1/2 (Thr202/Tyr204) (p-ERK), Cul3, Keap1, HSP90 and Lamin A/C antibodies were all from Cell Signaling Technology (Danvers, MA); GAPDH was from Advanced Immunochemical Inc. (Long Beach, CA); Secondary antibodies were from DAKO.

N-acetyl-L-cysteine, ascorbic acid, tert-butylhydroquinone, camptothecin, etoposide and staurosporine were all obtained from Sigma (Dorset, UK).

### Cell treatments

Apoptosis was induced by treatment with 5 μM camptothecin for 24 hours, 1 μM etoposide for 48 hours, and 1 μM staurosporine for 3 hours. The percentage of apoptotic cells was measured by flow cytometry after double staining with Annexin-V (FITC) and Propidium Iodide (PI) using the FITC Annexin-V Apoptosis Detection Kit (BD Pharmingen, San Diego, CA) following the manufacturer’s instructions. Data were analyzed using Summit software. Caspase 3/7 activity was quantified by using Caspase-Glo 3/7 Assay from Promega (Southampton, UK). Cell viability was addressed by using CellTiter AQueousOne Solution Cell Proliferation Assay (Promega), a colorimetric method based on the reduction of a tetrazolium compound by NADPH or NADH produced by dehydrogenase enzymes in metabolically active cells. Levels of reduced glutathione (GSH) were quantified by using GSH-Glo Glutathione Assay (Promega) following the manufacturer’s instructions. Nuclear and cytoplasmic protein fractions were obtained by using NE-PER Nuclear and Cytoplasmic Extraction Kit (Pierce, Cramlington, UK). Experiments in hypoxia (1% or 5% oxygen concentration) were performed as previously described [31].

In the inhibition studies for the RAS-downstream signaling pathways, breast cancer cell lines MDA-MB-231 and MCF-7 were seeded onto 6-well plates and 24 hours later washed with PBS and subjected to free serum standard media. 24 hours later the cells were incubated with free serum standard media containing DMSO (Sigma) or the following chemicals: ERK kinases inhibitor U0126 (Calbiochem/MerckMillipore, Watford, UK); PI3K inhibitors LY294002 (Merck Biosciences Ltd, Nottingham, UK) and wortmannin (Cell Signaling Technology, Danvers, MA); and AKT inhibitor GSK690693 (Symansis, Timaru, NZ). After 16 hours incubation, RNA was collected and qRT-PCR was performed. Protein extracts were also collected for western-blot analysis.

### Quantitative real-time polymerase chain reaction (qRT-PCR)

Total RNA was extracted using RNEasy mini kit (Qiagen, Sussex, UK) and mRNA levels were quantified by qRT-PCR using Taqman Gene Expression Assays (Applied Biosystems, Paisley, UK). SYBR Green Master Mix (Applied Biosystems) was used with optimized forward (5′-GGAGTCAACGGATTTGGTCGTA-3′) and reverse (5′-GGCAACAATATCCACTTTACCAGAGT-3′) primers for GAPDH (reference gene) at final concentration of 300 nM. b-Actin (Applied Biosystems) was used as reference gene for the experiments in hypoxia. All experiments were performed at least by triplicate.

### Additional cell lines

Additional MSC lines with activated RAS or RAS-downstream effectors were generated by infection of MSC4 with the retroviral vector pBabe-hygro encoding constitutive active RAS (H-Ras^V12^), the membrane-targeted RAF-1 (Raf-CAAX), and myristoylated AKT (myrAKT), all kindly provided by Dr. Pablo Rodriguez-Viciana (UCL Cancer Institute, London, UK). Immortal human mammary epithelial cells (HMEC), obtained from Dr. Rodriguez-Viciana, were cultured in DMEM/F-12 containing 5% horse serum (Life Technologies/Invitrogen, Paisley, UK) and supplemented with EGF (20 ng/ml) (Peprotech, London, UK), hydrocortisone (500 ng/ml), cholera toxin (100 ng/ml) and insulin (10 μg/ml), all from Sigma (Dorset, UK). HMEC expressing different oncogene combinations were generated after infection with the same retroviral vectors used for the generation of MSC lines [31]. Breast cancer cell lines MDA-MB-231 and MCF-7 were cultured in DMEM containing 10% fetal bovine serum (Life Technologies/Invitrogen). Human umbilical vein endothelial cells (HUVEC) were obtained from Promocell (Heidelberg, Germany) and cultured with Endothelial Cell Growth Medium (Promocell) according to supplier’s instructions.

### Public transcriptome data

The expression level of Nrf2 in many types of cancer was compared using the Oncomine ( https://www.oncomine.org/resource/login.html) and The Cancer Genome Atlas (TCGA) ( https://genome-cancer.ucsc.edu) databases of cancer expression data. We downloaded the available expression data with clinical details for 15 types of cancer from TCGA ( https://tcga-data.nci.nih.gov/tcga) and 3 types of cancer from the NCBI gene expression omnibus (GEO) ( http://www.ncbi.nlm.nih.gov/geo), including 5 separate breast cancer study datasets. In total, for survival analyses we studied 16 distinct types of cancer. Details of each dataset, the number of samples with clinical details, the expression platform, and associated Pubmed IDs for the GEO datasets are in Additional file 13: Table S3.

### Gene expression microarray analysis

Generation of Gene Expression Microarrays (GEM) was previously described [31] and data were deposited in ArrayExpress database (accession no. E-MEXP-563). Gene Set Enrichment Analysis (GSEA) measures the enrichment of a gene set within a GEM experiment. The enrichment score (ES) is a metric of the skew of a gene set within the rank of genes sorted by their GEM expression difference. The significance of enrichment (q, or false discovery rate) is the proportion of true ES >1000 ES generated from random gene sets (of equal size) (e.g., proportion of ES^OBSERVED^ > ES^NULL^). Leading-edge genes are the subset that contributes most to the ES.

### Statistical analysis

For survival analysis we used the R survival package. To survey for potential association between gene expression and survival we categorized samples as below or above median expression for each gene and then calculated the log-rank (Mantel Cox) *P* value comparison between the groups. For KIRC, SKCM and PRAD-GSE21034 datasets with significant NFE2L2 log rank tests we also calculated the hazard ratio using the Cox proportional hazard model. Elsewhere data were analyzed using Student’s *t* test, Spearmans rho or log-rank test as appropriate for the analysis. Values are given as mean ± SD. All statistical tests were two-sided, and results were considered statistically significant when *P* < 0.05.

## Abbreviations

MSC: Mesenchymal stem cell; HMEC: Human mammary epithelial cells; HUVEC: Human umbilical vein endothelial cells; ROS: Reactive oxygen species; GSEA: Gene set enrichment analysis; ARE: Antioxidant response element; TCGA: The cancer genome atlas; GEO: Gene expression omnibus; AsA: Ascorbic acid; NAC: N-acetyl-L-cysteine; TBHQ: Tert-butylhydroquinone; GSH: Reduced glutathione; KIRC: Kidney renal clear cell carcinoma; PRAD: Prostate adenocarcinoma; SKCM: Skin cutaneous melanoma.

## Competing interests

The authors declare that they have no competing interests.

## Authors’ contributions

JM Funes designed and performed the experiments, except the generation of HMEC lines. RK generated and characterized the HMEC lines expressing different oncogenes. JM Flanagan carried out the GSEA for the MSC lines and the TCGA- and Oncomine-based data analyses of Nrf2 expression. SH performed the survival and correlation analysis with the cancer expression datasets from GEO and TCGA. MR and BP conducted and analyzed the *in vivo* tumor growth experiments in mice. JM Funes, SM and CB conceived, designed and wrote the paper. All authors read and approved the final manuscript.

## Supplementary Material

Additional file 1: Table S1 A list of genes involved in ROS metabolism was generated based on a published gene list (see Supplementary Table Four from [34]). Genes included in Sabiosciences ( http://www.sabiosciences.com) Oxidative Stress Array databases, and other genes known to be involved in production or scavenging of ROS were also included in this list. Table shows all the Affymetrix probes for these genes (UNIQID) as well as the gene names, accession numbers and chromosome localization. Click here for file

Additional file 2: Figure S1 Percentage of viable MDA-MB-231 cells (A) and MCF-7 cells (B) after treatment with control DMSO or increasing concentrations of U0126, LY294002, GSK690693, and wortmannin. Cells were incubated with the inhibitors for 16 hours in serum free medium and viability was addressed by using CellTiter AQueousOne Solution Cell Proliferation Assay (Promega) following the manufacturer’s instructions. # indicates the concentration of the inhibitors used in the assay. Click here for file

Additional file 3: Figure S2 Expression of Cul3 and Keap1 does not significantly increase during the process of MSC transformation. Western-blot shows Cul3 and Keap1 protein levels. Membrane was also probed with polyclonal antibodies for Nrf2, and monoclonal antibodies for NQO1. Actin was used as a loading control. Click here for file

Additional file 4: Figure S3 Nrf2 stabilization by treatment with TBHQ reduces tHMEC *in vitro* transformation. (A) tHMEC treated with 10 μM TBHQ stabilizes Nrf2 and induces Nrf2-downstream gene NQO1. Membrane was probed with polyclonal antibodies for Nrf2, and monoclonal antibodies for NQO1. HSP90 was used as a loading control. (B) Soft agarose colonies counted in triplicate wells of tHMEC treated with vehicle only or 10 μM TBHQ after 12 days in culture. *P* value is *P < 0.05. Click here for file

Additional file 5: Figure S4 Nrf2 sensitizes transformed cells to apoptosis induced by the DNA-damaging agent camptothecin. (A) Geometrical mean (Geomean) fluorescence intensity measured by flow cytometry of transformed cells expressing empty vector or Nrf2 after 24 hours treatment with either vehicle or 5 μM camptothecin. The data show the mean ± S.D. from five independent experiments. A representative experiment is shown in panel (B). *P* value is *P < 0.05. Click here for file

Additional file 6: Figure S5 (A) Treatment with the DNA-damaging agent etoposide (an inhibitor of class II topoisomerase) sensitizes Nrf2-over-expressing cells to apoptosis measured by increased levels of cleaved PARP protein. (B) Likewise, cells over-expressing Nrf2 showed increased cytotoxicity following treatment with the kinase inhibitor staurosporine, as measured by increased caspase 3 and 7 activity (top panel) and by accumulation of cleaved PARP protein (bottom panel). Membranes were probed with polyclonal antibodies for cleaved PARP and Nrf2, and GAPDH served as loading control. *P* value is *P < 0.05. Click here for file

Additional file 7: Figure S6 qRT-PCR experiment showing the relative mRNA expression of the ADM gene in hypoxic conditions (5% O_2_ and 1% O_2_). To calculate the ADM fold induction relative to normoxia the mRNA levels for cells grown at 21% O_2_ were set as 1. *P* values are *P < 0.05 and ***P < 0.0005. Click here for file

Additional file 8: Figure S7 Nrf2 was found down-regulated in the majority of human tumors, as compared to normal tissues (right of vertical dotted line). For this analysis, 1003 tumors versus 485 normal or benign samples were included. Data are presented as log-transformed, median centered per array, and standard deviation normalized to 1 per array. Only cancers in which statistically significant expression is detected between normal and matched tumors are presented (*P* < 0.0001). These data are taken from Oncomine database ( https://www.oncomine.org/resource/login.html). Click here for file

Additional file 9: Figure S8 Expression of Nrf2 downstream genes NQO1 and GCLC is significantly down-regulated in kidney and prostate tumors respectively, whereas GCLM is found down-regulated in lung adenocarcinoma and breast tumors, but up-regulated in melanoma and prostate tumors. Data are presented as log-transformed, and normalized by subtracting the mean across all arrays for each gene. The number of samples analyzed (N) and the type of tumors are indicated. *P* values for those tumors where Nrf2 is down-regulated (in blue) or up-regulated (in red) are also shown. These data are taken from TCGA database ( https://genome-cancer.ucsc.edu). Click here for file

Additional file 10: Figure S9 The expression of the Nrf2 inhibitor Keap1 does not significantly change between normal and tumors samples, except for lymphoma tumors where Keap1 expression was found up-regulated (*P* = 4.7E-6) when compared to normal tissue. Data are presented as log-transformed, and normalized by subtracting the mean across all arrays for each gene. *P* values are also shown. These data are taken from TCGA database ( https://genome-cancer.ucsc.edu). Click here for file

Additional file 11: Table S2 We studied the relationship between survival and expression of Nrf2 (NFE2L2), GCLM, NQO1 and GCLC in a wide selection of cancers datasets acquired from GEO and TCGA databases. Samples were categorized as below or above median expression for each gene. Table shows the *P* values calculated from the log rank (Mantel Cox) test. Data in blue indicates that lower expression is correlated with poorer survival, and data in red indicates that higher expression is correlated with poorer survival. Click here for file

Additional file 12: Figure S10 Heatmaps show down-regulation of Nrf2 (NFE2L2) and selected antioxidants and Nrf2-downstream genes following oncogenic transformation of mouse and human cells. Red indicates up-regulation, and blue down-regulation from the mean. Arrow indicates Nrf2. (A) Results were obtained from mouse embryonic fibroblast (MEF) transformed with the adenovirus oncoprotein E1A and H-Ras^V12^ (tMEF). Microarray data were obtained upon request from Dr. Juan Iovanna [47]. (B) Results were obtained from human primary diploid fibroblasts IMR90 (HF) transformed with five oncogenic hits including hTERT, MEK1 (MEK-ER), E6 and E7 from HPV-16, and ST antigen from SV40 [48] (GSE2487 DataSets). HF1 represent immortal human fibroblast expressing hTERT and an empty vector, and tHF represent transformed human fibroblast expressing the five oncogenic hits described above. Click here for file

Additional file 13: Table S3 Transcriptome datasets. Table shows the details of each cancer dataset, the number of samples with clinical details, the expression platform, and associated Pubmed IDs for the GEO datasets. Click here for file
