# Supplementary material for: Oncogenic transformation of mesenchymal stem cells decreases Nrf2 expression favoring in vivo tumor growth and poorer survival
Source: Mol Cancer. 2014 Feb 3;13:20. doi: 10.1186/1476-4598-13-20 (PMC4015761; doi:10.1186/1476-4598-13-20)
Supplement: Additional file 1: Table S1 — A list of genes involved in ROS metabolism was generated based on a published gene list (see Supplementary Table Four from [34]). Genes included in Sabiosciences ( http://www.sabiosciences.com) Oxidative Stress Array databases, and other genes known to be involved in production or scavenging of ROS were also included in this list. Table shows all the Affymetrix probes for these genes (UNIQID) as well as the gene names, accession numbers and chromosome localization. [file 1476-4598-13-20-S1.pdf]

Supplementary Table S1

UNIQID	NAME	Gene mane	Accession	Chromosome
200736_s_at	GPX1	glutathione peroxidase 1	NM_000581	3
201106_at	GPX4	glutathione peroxidase 4 (phospholipid hydroperoxidase)	NM_002085	19
201348_at	GPX3	glutathione peroxidase 3 (plasma)	NM_002084	5
202831_at	GPX2	glutathione peroxidase 2 (gastrointestinal)	NM_002083	14
208028_s_at	GPX5	glutathione peroxidase 5 (epididymal androgen-related protein)	NM_003996	6
213170_at	GPX7	glutathione peroxidase 7	AA406605	1
214091_s_at	GPX3	glutathione peroxidase 3 (plasma)	AW149846	5
214648_at	GPX5	glutathione peroxidase 5 (epididymal androgen-related protein)	AI207120	6
215760_s_at	GPX4	strawberry notch homolog 2 (Drosophila)	AC005390	19
239595_at	GPX2	glutathione peroxidase 2 (gastrointestinal)	AA569032	14
205770_at	GSR	glutathione reductase	NM_000637	8
225609_at	GSR	glutathione reductase	AI888037	8
237402_at	GSR	NA	H05917	NA
208864_s_at	TXN	thioredoxin	AF313911	9
216609_at	TXN	thioredoxin	AF065241	9
209078_s_at	TXN2	thioredoxin 2	AF276920	22
206662_at	GLRX	glutaredoxin (thioltransferase)	NM_002064	5
209276_s_at	GLRX	glutaredoxin (thioltransferase)	AF162769	5
219933_at	GLRX2	glutaredoxin 2	NM_016066	1
223342_at	RRM2B	ribonucleotide reductase M2 B (TP53 inducible)	AB036063	8
201266_at	TXNRD1	thioredoxin reductase 1	NM_003330	12
210803_at	TXNRD2	thioredoxin reductase 2	AF201385	22
211177_s_at	TXNRD2	thioredoxin reductase 2	AB019695	22
221906_at	TXNRD3	thioredoxin reductase 3	BF513089	3
230880_at	TXNRD2	KIAA1652 protein	AW450772	22
231292_at	TXNRD1	EP300 interacting inhibitor of differentiation 3	AI964053	12
59631_at	TXNRD3	thioredoxin reductase 3	AI247566	3
201467_s_at	NQO1	NAD(P)H dehydrogenase, quinone 1	AI039874	16
201468_s_at	NQO1	NAD(P)H dehydrogenase, quinone 1	NM_000903	16
203814_s_at	NQO2	NAD(P)H dehydrogenase, quinone 2	NM_000904	6
210519_s_at	NQO1	NAD(P)H dehydrogenase, quinone 1	BC000906	16
200642_at	SOD1	superoxide dismutase 1, soluble	NM_000454	21
205236_x_at	SOD3	superoxide dismutase 3, extracellular	NM_003102	4
215078_at	SOD2	superoxide dismutase 2, mitochondrial	AL050388	6
215223_s_at	SOD2	superoxide dismutase 2, mitochondrial	W46388	6
216841_s_at	SOD2	superoxide dismutase 2, mitochondrial	X15132	6
201432_at	CAT	catalase	NM_001752	11
211922_s_at	CAT	catalase	AY028632	11
215573_at	CAT	catalase	AU147084	11
206418_at	NOX1	NADPH oxidase 1	NM_007052	X
207217_s_at	NOX1	NADPH oxidase 1	NM_013955	X
207380_x_at	NOX1	NADPH oxidase 1	NM_013954	X
210808_s_at	NOX1	NADPH oxidase 1	AF166327	X
230399_at	NOX1	NA	AI361034	NA
203922_s_at	CYBB	cytochrome b-245, beta polypeptide	AI308863	X
203923_s_at	CYBB	cytochrome b-245, beta polypeptide	NM_000397	X
217431_x_at	CYBB	cytochrome b-245, beta polypeptide	S67289	X

233538_s_at	CYBB	cytochrome b-245, beta polypeptide	AI203028	X
221089_at	NOX3	NADPH oxidase 3	NM_015718	6
219773_at	NOX4	NADPH oxidase 4	NM_016931	11
1553023_a_at	NOX5	NADPH oxidase, EF-hand calcium binding domain 5	NM_024505	15
220641_at	NOX5	NADPH oxidase, EF-hand calcium binding domain 5	NM_024505	15
229352_at	NOX5	NA	AA885360	NA
210301_at	XDH	xanthine dehydrogenase	U06117	2
241994_at	XDH	xanthine dehydrogenase	BG260086	2
205082_s_at	AOX1	aldehyde oxidase 1	AB046692	2
205083_at	AOX1	aldehyde oxidase 1	NM_001159	2
1560587_s_at	PRDX5	peroxiredoxin 5	AI718223	11
200844_s_at	PRDX6	peroxiredoxin 6	BE869583	1
200845_s_at	PRDX6	peroxiredoxin 6	NM_004905	1
201006_at	PRDX2	peroxiredoxin 2	NM_005809	19
201619_at	PRDX3	peroxiredoxin 3	NM_006793	10
201923_at	PRDX4	peroxiredoxin 4	NM_006406	X
208680_at	PRDX1	peroxiredoxin 1	L19184	1
209766_at	PRDX3	peroxiredoxin 3	AF118073	10
211658_at	PRDX2	peroxiredoxin 2	L19185	19
215067_x_at	PRDX2	peroxiredoxin 2	AU147942	19
222994_at	PRDX5	peroxiredoxin 5	AF197952	11
238951_at	PRDX6	NA	BF843343	NA
242751_at	PRDX6	NA	N55072	NA
39729_at	PRDX2	peroxiredoxin 2	L19185	19
203665_at	HMOX1	heme oxygenase (decycling) 1	NM_002133	22
202275_at	G6PD	glucose-6-phosphate dehydrogenase	NM_000402	X
38043_at	G6PD	family with sequence similarity 3, member A	X55448	X
201118_at	PGD	phosphogluconate dehydrogenase	NM_002631	1
1555037_a_at	IDH1	isocitrate dehydrogenase 1 (NADP+), soluble	BC012846	2
201193_at	IDH1	isocitrate dehydrogenase 1 (NADP+), soluble	NM_005896	2
242001_at	IDH1	isocitrate dehydrogenase 1 (NADP+), soluble	AA825652	2
242956_at	IDH1	isocitrate dehydrogenase 1 (NADP+), soluble	AA564352	2
210045_at	IDH2	isocitrate dehydrogenase 2 (NADP+), mitochondrial	AU151428	15
210046_s_at	IDH2	isocitrate dehydrogenase 2 (NADP+), mitochondrial	U52144	15
204058_at	ME1	malic enzyme 1, NADP(+)-dependent, cytosolic	AL049699	6
204059_s_at	ME1	malic enzyme 1, NADP(+)-dependent, cytosolic	NM_002395	6
211204_at	ME1	malic enzyme 1, NADP(+)-dependent, cytosolic	L34035	6
213555_at	ME1	RWD domain containing 2A	AL049699	6
240788_at	ME1	NA	AI076834	NA
1562612_at	ME2	NA	BC020933	NA
209397_at	ME2	malic enzyme 2, NAD(+)-dependent, mitochondrial	BC000147	18
210153_s_at	ME2	malic enzyme 2, NAD(+)-dependent, mitochondrial	M55905	18
210154_at	ME2	malic enzyme 2, NAD(+)-dependent, mitochondrial	M55905	18
204663_at	ME3	malic enzyme 3, NADP(+)-dependent, mitochondrial	NM_006680	11
231295_at	ME3	malic enzyme 3, NADP(+)-dependent, mitochondrial	BE326351	11
202783_at	NNT	nicotinamide nucleotide transhydrogenase	U40490	5
202784_s_at	NNT	nicotinamide nucleotide transhydrogenase	NM_012343	5
215278_at	NNT	NA	AF052090	NA
238530_at	NNT	nicotinamide nucleotide transhydrogenase	BF695396	5
244531_at	NNT	nicotinamide nucleotide transhydrogenase	BE501279	5

200758_s_at	NFE2L1	nuclear factor (erythroid-derived 2)-like 1	AI361227	17
200759_x_at	NFE2L1	nuclear factor (erythroid-derived 2)-like 1	NM_003204	17
214179_s_at	NFE2L1	nuclear factor (erythroid-derived 2)-like 1	H93013	17
1567013_at	NFE2L2	nuclear factor (erythroid-derived 2)-like 2	AF323119	2
1567014_s_at	NFE2L2	nuclear factor (erythroid-derived 2)-like 2	AF323119	2
1567015_at	NFE2L2	nuclear factor (erythroid-derived 2)-like 2	AF323119	2
201146_at	NFE2L2	nuclear factor (erythroid-derived 2)-like 2	NM_006164	2
236213_at	NFE2L2	NA	AI809760	NA
203925_at	GCLM	glutamate-cysteine ligase, modifier subunit	NM_002061	1
234986_at	GCLM	NA	AA630626	NA
236140_at	GCLM	glutamate-cysteine ligase, modifier subunit	AI753488	1
1555330_at	GCLC	glutamate-cysteine ligase, catalytic subunit	BC022487	6
202922_at	GCLC	glutamate-cysteine ligase, catalytic subunit	BF676980	6
202923_s_at	GCLC	glutamate-cysteine ligase, catalytic subunit	NM_001498	6
201415_at	GSS	glutathione synthetase	NM_000178	20
211630_s_at	GSS	glutathione synthetase	L42531	20
209918_at	GGT2	NA	J05235	NA
207131_x_at	GGT1	gamma-glutamyltransferase 1	NM_013430	22
208284_x_at	GGT1	gamma-glutamyltransferase 1	NM_013421	22
209919_x_at	GGT1	gamma-glutamyltransferase 1	L20490	22
211417_x_at	GGT1	gamma-glutamyltransferase 1	L20493	22
215603_x_at	GGT1	NA	AI344075	NA
233837_at	GGT1	gamma-glutamyltransferase 1	AU147678	22
205970_at	MT3	metallothionein 3	NM_005954	16
203522_at	CCS	copper chaperone for superoxide dismutase	NM_005125	11
203028_s_at	CYBA	cytochrome b-245, alpha polypeptide	NM_000101	16
200748_s_at	FTH1	ferritin, heavy polypeptide 1	NM_002032	11
214211_at	FTH1	ferritin, heavy polypeptide 1	AA083483	11
237293_at	FTH1	NA	AI808844	NA
243702_at	FTH1	NA	AA722627	NA
212788_x_at	FTL	ferritin, light polypeptide	BG537190	19
213187_x_at	FTL	ferritin, light polypeptide	BG538564	19
210852_s_at	AASS	aminoadipate-semialdehyde synthase	AF229180	7
214829_at	AASS	aminoadipate-semialdehyde synthase	AK023446	7
1555284_at	ALS2	amyotrophic lateral sclerosis 2 (juvenile)	BC029174	2
1560742_at	ALS2	NA	AI985407	NA
1560743_a_at	ALS2	NA	AI985407	NA
226291_at	ALS2	amyotrophic lateral sclerosis 2 (juvenile)	AB046783	2
232184_at	ALS2	amyotrophic lateral sclerosis 2 (juvenile)	AK023024	2
203381_s_at	APOE	apolipoprotein E	N33009	19
203382_s_at	APOE	apolipoprotein E	NM_000041	19
212874_at	APOE	NA	AI358867	NA
212883_at	APOE	NA	AI358867	NA
212884_x_at	APOE	apolipoprotein E	AI358867	19
214627_at	EPX	eosinophil peroxidase	X14346	17
207347_at	ERCC6	excision repair cross-complementing rodent repair deficiency, complementation group 6	NM_000124	10
1557030_at	GAB1	GRB2-associated binding protein 1	BC030751	4
1560382_at	GAB1	GRB2-associated binding protein 1	AK074381	4
207112_s_at	GAB1	GRB2-associated binding protein 1	NM_002039	4
214987_at	GAB1	GRB2-associated binding protein 1	AL049449	4

225998_at	GAB1	GRB2-associated binding protein 1	AK022142	4
226002_at	GAB1	GRB2-associated binding protein 1	AK022142	4
229114_at	GAB1	GRB2-associated binding protein 1	AW237741	4
242572_at	GAB1	NA	BF435438	NA
203948_s_at	MPO	myeloperoxidase	J02694	17
203949_at	MPO	myeloperoxidase	NM_000250	17
1556761_at	MSRA	NA	AI057305	NA
1556762_a_at	MSRA	NA	AI057305	NA
1559586_at	MSRA	hypothetical protein LOC728868	BE504795	8
219281_at	MSRA	methionine sulfoxide reductase A	NM_012331	8
233393_at	MSRA	NA	AU146871	NA
234435_at	MSRA	NA	AL049349	NA
234837_at	MSRA	NA	AL049349	NA
237341_at	MSRA	NA	H98180	NA
237798_at	MSRA	NA	AI074612	NA
240031_at	MSRA	methionine sulfoxide reductase A	AA994467	8
209949_at	NCF2	neutrophil cytosolic factor 2	BC001606	1
235329_at	NOXO1	NADPH oxidase organizer 1	AW083983	16
1554103_at	PPP1R15B	NA	BC009873	NA
224692_at	PPP1R15B	protein phosphatase 1, regulatory (inhibitor) subunit 15B	BF796046	1
201300_s_at	PRNP	prion protein	NM_000311	20
215707_s_at	PRNP	prion protein	AV725328	20
222106_at	PRNP	prion protein 2 (dublet)	AL133396	20
210342_s_at	TPO	thyroid peroxidase	M17755	2
201008_s_at	TXNIP	thioredoxin interacting protein	AA812232	1
201009_s_at	TXNIP	thioredoxin interacting protein	AI439556	1
201010_s_at	TXNIP	thioredoxin interacting protein	NM_006472	1
207349_s_at	UCP3	uncoupling protein 3 (mitochondrial, proton carrier)	NM_022803	11
219827_at	UCP3	uncoupling protein 3 (mitochondrial, proton carrier)	NM_003356	11
205672_at	XPA	xeroderma pigmentosum, complementation group A	NM_000380	9
202723_s_at	FOXO1A	forkhead box O1	AW117498	13
202724_s_at	FOXO1A	forkhead box O1	NM_002015	13
228484_s_at	FOXO1A	forkhead box O1	AI472322	13
232882_at	FOXO1A	NA	AA079839	NA
239728_at	FOXO1A	NA	AA810830	NA
241665_x_at	FOXO1A	NA	AI307430	NA
241667_x_at	FOXO1A	NA	AI820891	NA
1569477_at	FOXO3A	NA	BC025999	NA
204131_s_at	FOXO3A	forkhead box O3	N25732	6
204132_s_at	FOXO3A	NA	NM_001455	NA
210655_s_at	FOXO3A	NA	AF041336	NA
224889_at	FOXO3A	forkhead box O3	BE888885	6
224891_at	FOXO3A	forkhead box O3	AV725666	6
233629_at	FOXO3A	NA	AK026286	NA
233674_at	FOXO3A	NA	AK026286	NA
239923_at	FOXO3A	NA	AI056872	NA
242320_at	FOXO3A	NA	AI435586	NA
205451_at	MLLT7	forkhead box O4	NM_005938	X
218498_s_at	ERO1L	ERO1-like (S. cerevisiae)	NM_014584	14
222646_s_at	ERO1L	ERO1-like (S. cerevisiae)	AW268365	14

225750_at	ERO1L	NA	BE966748	NA
220012_at	ERO1LB	ERO1-like beta (S. cerevisiae)	NM_019891	1
231944_at	ERO1LB	ERO1-like beta (S. cerevisiae)	AL045717	1
202201_at	BLVRB	biliverdin reductase B (flavin reductase (NADPH))	NM_000713	19
209531_at	GSTZ1	glutathione transferase zeta 1	BC001453	14
232913_at	GSTZ1	transmembrane emp24 protein transport domain containing 8	AC007954	14
202646_s_at	UNR	cold shock domain containing E1, RNA-binding	AA167775	1
219939_s_at	UNR	cold shock domain containing E1, RNA-binding	NM_007158	1
222975_s_at	UNR	cold shock domain containing E1, RNA-binding	AI423180	1
1553572_a_at	CYGB	cytoglobin	NM_134268	17
1570410_at	CYGB	cytoglobin	BC018822	17
226632_at	CYGB	cytoglobin	AL513673	17
1565795_at	DUOX1	dual oxidase 1	BI768821	15
215800_at	DUOX1	dual oxidase 1	AL137592	15
219597_s_at	DUOX1	dual oxidase 1	NM_017434	15
219727_at	DUOX2	dual oxidase 2	NM_014080	15
1553028_at	GPR156	G protein-coupled receptor 156	NM_153002	3
210682_at	LPO	lactoperoxidase	U39573	17
201403_s_at	MGST3	microsomal glutathione S-transferase 3	NM_004528	1
233307_x_at	MGST3	NA	AK000073	NA
244122_at	MGST3	microsomal glutathione S-transferase 3	AA129724	1
214735_at	PIP3-E	interaction protein for cytohesin exchange factors 1	AW166711	6
241188_at	PIP3-E	NA	BF223340	NA
243032_at	PIP3-E	NA	AI476542	NA
205127_at	PTGS1	prostaglandin-endoperoxide synthase 1 (prostaglandin G/H synthase and cyclooxygenase)	NM_000962	9
205128_x_at	PTGS1	prostaglandin-endoperoxide synthase 1 (prostaglandin G/H synthase and cyclooxygenase)	NM_000962	9
215813_s_at	PTGS1	prostaglandin-endoperoxide synthase 1 (prostaglandin G/H synthase and cyclooxygenase)	S36219	9
238669_at	PTGS1	prostaglandin-endoperoxide synthase 1 (prostaglandin G/H synthase and cyclooxygenase)	BE613133	9
240171_at	PTGS1	NA	AW206099	NA
1554997_a_at	PTGS2	prostaglandin-endoperoxide synthase 2 (prostaglandin G/H synthase and cyclooxygenase)	AY151286	1
204748_at	PTGS2	prostaglandin-endoperoxide synthase 2 (prostaglandin G/H synthase and cyclooxygenase)	NM_000963	1
212012_at	PXDN	peroxidasin homolog (Drosophila)	BF342851	2
212013_at	PXDN	peroxidasin homolog (Drosophila)	D86983	2
241942_at	PXDNL	peroxidasin homolog (Drosophila)-like	AA927870	8
1556043_a_at	TTN	NA	AK093733	NA
1557994_at	TTN	titin	BF574523	2
1561230_at	TTN	NA	BC043155	NA
208195_at	TTN	titin	NM_003319	2
240793_at	TTN	titin	BF224054	2
241791_at	TTN	titin	BF825274	2
242679_at	TTN	NA	AU127120	NA
242729_at	TTN	NA	BE551384	NA
242771_at	TTN	titin	AU149821	2
244839_at	TTN	titin	AW975934	2
1565228_s_at	ALB	albumin	D16931	4
211298_s_at	ALB	albumin	AF116645	4
214837_at	ALB	albumin	M12523	4
214842_s_at	ALB	NA	M12523	NA

223209_s_at	SELS	selenoprotein S	AF328864	15
1552657_a_at	TXNDC2	thioredoxin domain containing 2 (spermatozoa)	NM_032243	18
224168_at	TXNDC2	thioredoxin domain containing 2 (spermatozoa)	AL136742	18
204961_s_at	NCF1	NA	NM_000265	NA
214084_x_at	NCF1	neutrophil cytosolic factor 1C pseudogene	AW072388	7
207206_s_at	ALOX12	arachidonate 12-lipoxygenase	NM_000697	17
229833_at	ALOX12	NA	BF507533	NA
236433_at	ALOX12	NA	AW450397	NA
201065_s_at	GTF2I	NA	NM_001518	NA
210891_s_at	GTF2I	NA	AF035737	NA
210892_s_at	GTF2I	general transcription factor IIi	BC004472	7
215569_at	GTF2I	NA	AC004883	NA
229896_at	GTF2I	general transcription factor IIi	H41907	7
210037_s_at	NOS2A	nitric oxide synthase 2, inducible	L24553	17
224909_s_at	PREX1	phosphatidylinositol-3,4,5-trisphosphate-dependent Rac exchange factor 1	BF308645	20
224925_at	PREX1	phosphatidylinositol-3,4,5-trisphosphate-dependent Rac exchange factor 1	AL445192	20
220811_at	PRG3	proteoglycan 3	NM_006093	11
201848_s_at	BNIP3	BCL2/adenovirus E1B 19kDa interacting protein 3	U15174	10
201849_at	BNIP3	BCL2/adenovirus E1B 19kDa interacting protein 3	NM_004052	10
243117_at	BNIP3	NA	AL038973	NA
209368_at	EPHX2	epoxide hydrolase 2, cytoplasmic	AF233336	8
203466_at	MPV17	MpV17 mitochondrial inner membrane protein	NM_002437	2
214199_at	SFTPD	surfactant protein D	NM_003019	10
206423_at	ANGPTL7	angiopoietin-like 7	NM_021146	1
1405_i_at	CCL5	chemokine (C-C motif) ligand 5	M21121	17
1555759_a_at	CCL5	chemokine (C-C motif) ligand 5	AF043341	17
200862_at	DHCR24	24-dehydrocholesterol reductase	NM_014762	1
201041_s_at	DUSP1	dual specificity phosphatase 1	NM_004417	5
201044_x_at	DUSP1	dual specificity phosphatase 1	AA530892	5
226578_s_at	DUSP1	dual specificity phosphatase 1	AW024420	5
202580_x_at	FOXM1	forkhead box M1	NM_021953	12
205900_at	KRT1	keratin 1	NM_006121	12
207256_at	MBL2	mannose-binding lectin (protein C) 2, soluble (opsonic defect)	NM_000242	10
219786_at	MTL5	metallothionein-like 5, testis-specific (tesmin)	NM_004923	11
238246_at	MTL5	metallothionein-like 5, testis-specific (tesmin)	BE467611	11
241466_at	MTL5	NA	AI275776	NA
206197_at	NME5	non-metastatic cells 5, protein expressed in (nucleoside-diphosphate kinase)	NM_003551	5
204766_s_at	NUDT1	nudix (nucleoside diphosphate linked moiety X)-type motif 1	NM_002452	7
228231_at	NUDT1	NA	BE221804	NA
243135_x_at	NUDT1	NA	R26456	NA
218197_s_at	OXR1	oxidation resistance 1	NM_018002	8
222553_x_at	OXR1	oxidation resistance 1	AL541048	8
223879_s_at	OXR1	oxidation resistance 1	AF309387	8
238408_at	OXR1	NA	AW086258	NA
238409_x_at	OXR1	oxidation resistance 1	AW086261	8
202696_at	OXSR1	oxidative-stress responsive 1	NM_005109	3
208690_s_at	PDLIM1	PDZ and LIM domain 1	BC000915	10
213389_at	PNKP	zinc finger protein 592	BF508616	15
218961_s_at	PNKP	polynucleotide kinase 3'-phosphatase	NM_007254	19
218286_s_at	RNF7	ring finger protein 7	NM_014245	3

224394_at	RNF7	ring finger protein 7	AF312226	3
224395_s_at	RNF7	ring finger protein 7	AF312226	3
224439_x_at	RNF7	ring finger protein 7	BC005966	3
239820_at	RNF7	ring finger protein 7	AW593225	3
219416_at	SCARA3	scavenger receptor class A, member 3	NM_016240	8
223842_s_at	SCARA3	scavenger receptor class A, member 3	AB007830	8
223843_at	SCARA3	scavenger receptor class A, member 3	AB007830	8
201427_s_at	SEPP1	selenoprotein P, plasma, 1	NM_005410	5
229620_at	SEPP1	selenoprotein P, plasma, 1	BE856597	5
231669_at	SEPP1	selenoprotein P, plasma, 1	AV653290	5
242519_at	SEPP1	NA	BF432331	NA
213837_at	SGK2	l(3)mbt-like (Drosophila)	Z98752	20
220357_s_at	SGK2	serum/glucocorticoid regulated kinase 2	NM_016276	20
230573_at	SGK2	serum/glucocorticoid regulated kinase 2	AI631895	20
1558331_at	SIRT2	sirtuin (silent mating type information regulation 2 homolog) 2 (S. cerevisiae)	BG722779	19
220605_s_at	SIRT2	sirtuin (silent mating type information regulation 2 homolog) 2 (S. cerevisiae)	NM_012237	19
201314_at	STK25	serine/threonine kinase 25 (STE20 homolog, yeast)	NM_006374	2
225652_at	STK25	NA	AA046941	NA
230768_at	STK25	NA	BE672541	NA
237050_at	STK25	NA	AW207725	NA
242857_at	STK25	NA	AA748613	NA
242932_at	STK25	NA	AW292329	NA
203454_s_at	ATOX1	ATX1 antioxidant protein 1 homolog (yeast)	NM_004045	5
